# Study on Propagation Depth of Ultrasonic Longitudinal Critically Refracted (LCR) Wave

**DOI:** 10.3390/s20195724

**Published:** 2020-10-08

**Authors:** Yongmeng Liu, Enxiao Liu, Yuanlin Chen, Xiaoming Wang, Chuanzhi Sun, Jiubin Tan

**Affiliations:** 1 Center of Ultra-Precision Optoelectronic Instrument Engineering, Harbin Institute of Technology, Harbin 150080, China; lym@hit.edu.cn (Y.L.); 18b301002@stu.hit.edu.cn (E.L.); 19S001068@stu.hit.edu.cn (Y.C.); czsun@hit.edu.cn (C.S.); jbtan@hit.edu.cn (J.T.); 2 Key Lab of Ultra-Precision Intelligent Instrumentation Engineering (Harbin Institute of Technology), Ministry of Industry and Information Technology, Harbin 150080, China

**Keywords:** aero-engine rotor assembly, LCR wave, propagation depth, propagation time extension

## Abstract

The accurate measurement of stress at different depths in the end face of a high-pressure compressor rotor is particularly important, as it is directly related to the assembly quality and overall performance of aero-engines. The ultrasonic longitudinal critically refracted (LCR) wave is sensitive to stress and can measure stress at different depths, which has a prominent advantage in stress non-destructive measurements. In order to accurately characterize the propagation depth of LCR waves and improve the spatial resolution of stress measurement, a finite element model suitable for the study of LCR wave propagation depths was established based on a wave equation and Snell law, and the generation and propagation process of LCR waves are analyzed. By analyzing the blocking effect of grooves with different depths on the wave, the propagation depth of the LCR wave at seven specific frequencies was determined in turn. On this basis, the LCR wave propagation depth model is established, and the effects of wedge materials, piezoelectric element diameters, and excitation voltages on the propagation depth of LCR waves are discussed. This study is of great significance to improve the spatial resolution of stress measurements at different depths in the end face of the aero-engine rotor.

## 1. Introduction

The core engine system is composed of multi-stage rotors. The superposition of residual stress and assembly stress makes the distribution of stress fields at different positions and depths of the rotor components more complicated. The assembly quality of rotors at all levels has a great impact on the performance of aero-engines [1,2,3]. The unevenly distributed stress field will have a significant impact on the fatigue strength and structural deformation of the rotor, which will lead to the coaxiality error of the assembled rotor exceeding the standard. In the case of high-speed rotation, the imbalance response caused by the coaxiality error will be amplified, resulting in engine vibration and causing friction between the blade and the casing [4]. According to statistics, more than 70% of aero-engine faults are caused by vibrations and about 20% of aero-engine faults are caused by friction [5,6]. Therefore, it is necessary to realize the precise control of stress at different depths in the rotor end face through accurate measurements [7,8,9] and then improve the uniformity of the stress field distribution to reduce the coaxiality error of the assembled rotor, so as to improve the safety and reliability of the aero-engine.

The measurement of stress includes three kinds of methods: non-destructive, micro-damage, and damage [10,11,12,13,14]. Both damage and micro-damage measurement methods can cause damage to the measured part and these damages are fatal to the finished parts or the workpieces in service. Therefore, the damage and micro-damage methods cannot effectively reflect the stress state of the measured parts. Moreover, they cannot measure or monitor the stress in a service state in real time. The non-destructive measurement method gives information on the magnitude, direction, and position of stress without damaging or affecting the performance of the measured part. Among the non-destructive measurement methods, the ultrasonic method has many advantages such as no radiation damage to the human body, a high spatial resolution, and a large range of measurement depths [15,16,17,18,19], which has always been the focus of scholars’ research. Ultrasonic stress measurement mainly includes longitudinal and transverse wave methods, the surface wave method, guided wave method, non-linear method, and longitudinal critically refracted (LCR) wave method.

Compared with other ultrasonic stress measurement methods, the LCR wave method is sensitive to the stress field and is less affected by the effect of material structures. It has outstanding advantages in the non-destructive measurement of residual stress. Therefore, the LCR wave method is currently the focus of research in various countries and the main development direction of ultrasonic stress measurement in the future [20,21].

In terms of the simulation study of the sound field characteristics of LCR waves, Pei [22] used the finite element method to simulate and study the LCR waves of electromagnetic ultrasonic transducers when measuring residual stress. Chaki [23] conducted a numerical simulation and experimental analysis of the LCR wave beam profile in a homogeneous and isotropic elastic solid medium. Through the study, the composition of the refracted sound field and the energy distribution of the LCR wave at different incident angles were obtained. The results of this study provide useful information for optimizing the incident angle of sound waves to obtain the optimal excitation energy of LCR waves. In the application study, Qozam uses the LCR wave to measure the welding stress of P355 steel. First, the acoustoelastic constants of the tissues at different distances from the weld were calibrated separately. Then, the stress of different tissues were measured, respectively. The measured stress distribution is very consistent with the data obtained by the blind hole method [24]. Yashar studied the LCR wave method in the axial and hoop stress measurement of austenitic stainless steel tubes. The LCR waves of different frequencies were used to measure the stress at different depths. Finally, the accuracy of the measurement was verified by a finite element simulation and blind hole method [25].

The accurate measurement of the propagation depth of LCR waves at different frequencies is a prerequisite for improving the spatial resolution of stress measurement and is essential for obtaining the true stress distribution state at different depths within the measured part. However, there is no definite theoretical formula for the relationship between the propagation depth of LCR waves and frequency, and it is currently mainly measured by the experiment in [26]. The measurement method is to mill a groove of a certain depth between the transmitting and receiving transducers on the measured part to hinder the propagation of LCR waves. The propagation depth of the LCR wave is determined by analyzing the received signals at different groove depths.

There are some problems in measuring the propagation depth of LCR waves by experiment:

(a) When milling the groove, stress will concentrate around the groove. The stress will change the propagation velocity of the ultrasonic wave, so the time of receiving the LCR wave signal may be affected by stress.

(b) When acquiring LCR wave signals at different groove depths, the repeated installation of ultrasonic transmitting and receiving transducers makes it difficult to keep the coupling state consistent. The change of coupling state will lead to the delay or advancement of the received signal. The propagation velocity of the ultrasonic wave in the most commonly used couplant is 1620 m/s. If the thickness of the couplant changes by 1 μm, a propagation time difference of 0.62 ns will be caused. Therefore, the change of couplant thickness has a great influence on the measurement of the propagation depth of LCR waves.

(c) The milled groove inevitably has dimensional errors, which will affect the measurement accuracy of the propagation depth of the LCR wave.

All the factors above will lead to an inaccurate measurement of the propagation depth of LCR waves. Only by obtaining an accurate LCR wave propagation depth model can the stress of different depths be measured more accurately. Then, the stress distribution in different depths in the end face of the rotor parts can be obtained and the corresponding control measures can be taken. To overcome the shortcomings of the experimental method to study the propagation depth of LCR waves, an ideal finite element numerical model is established to simulate the propagation depth of LCR waves. By this method, the influence of factors such as stress concentration, coupling state error, and machining error can be eliminated. The theoretical formula including frequency is established to solve the problem of the lack of an accurate LCR wave propagation depth model, so as to improve the measurement accuracy of LCR wave propagation depth. Thus, the spatial resolution of stress measurement at different depths in the end face of the aero-engine rotor parts can be improved.

The novelty of this study is to combine the idea of the groove method to determine the propagation depth of LCR wave with the advantages of a finite element simulation, thus providing an accurate method to determine the propagation depth of LCR waves. Through this method, the propagation depth of LCR waves in different materials can be studied, which provides a theoretical and technical reference for the measurement of stress at different depths under the surface of different materials. At the same time, changing the wedge material, piezoelectric element diameter, and excitation voltage parameters to study the change of LCR wave propagation depth is conducive to an accurate measurement and control of LCR wave propagation depth, and also guides the selection of ultrasonic stress measurement devices and their parameters.

## 2. Principle of LCR Wave Measuring Stress at Different Depths

**Figure 1:**
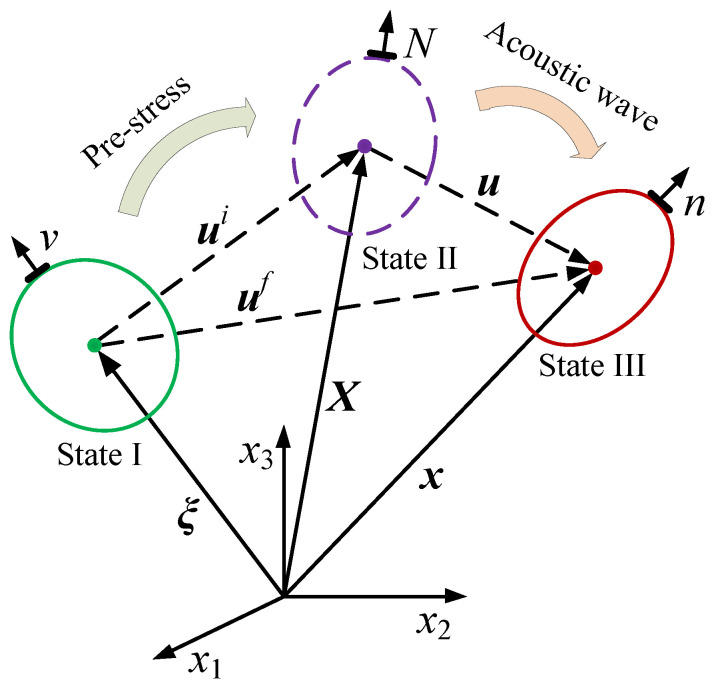
Coordinate system of deformed object.

The state of the solid material without stress and strain is called the natural state (state I), as shown in Figure 1. The state of the solid material when it has been deformed or is under a certain load is called the pre-deformed state (state II). A small acoustic disturbance is superimposed on the pre-deformed solid material to further deform the material to the final state—that is, the ultrasonic detection state (state III) [27,28].

In the initial coordinates, considering the influence of the stress on the medium and the attenuation of ultrasonic propagation, the equation of the elastic wave in the solid material is:

(1) $$\left(\delta_{IK}t_{JK}^{i}+C_{IJKL}\right)\frac{\partial^{2}u_{K}}{\partial X_{J}\partial X_{L}}-\gamma \frac{\partial u_{I}}{\partial t}+F=\rho_{0}\left(1-\varepsilon_{NN}^{i}\right)\frac{\partial^{2}u_{I}}{\partial t^{2}}$$

where *δ_IK_* is the Kronecker delta function, $\varepsilon_{NN}^{i}$ is the pre-strain tensor, *ρ*_0_ is the density of the solid material in natural coordinates, *γ* is the sound attenuation coefficient in the solid material, *F* is the external force, and *C_IJKL_*(*I*, *J*, *K*, *L* = 1, 2, 3) is the stiffness coefficient matrix of the material. 

**Figure 2:**
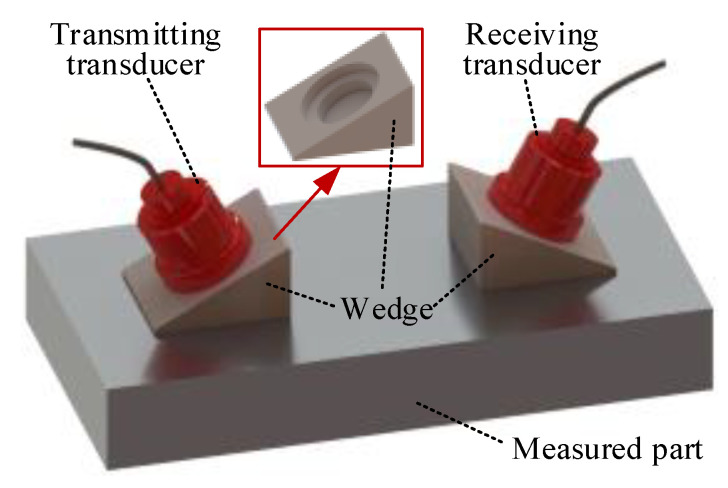
Longitudinal critically refracted (LCR) wave excitation and reception device and wedge structure.

When an LCR wave is used to measure stress, the wedge should be designed to allow the ultrasonic transducer to be installed obliquely. The angle of the inclined plane of the wedge is the first critical angle calculated according to Snell’s law. There is a circular groove on the inclined plane for installing the ultrasonic transducer, as shown in Figure 2. Polymethyl methacrylate (PMMA) and polystyrene (PS) are commonly used materials for making LCR wave wedges.

**Figure 3:**
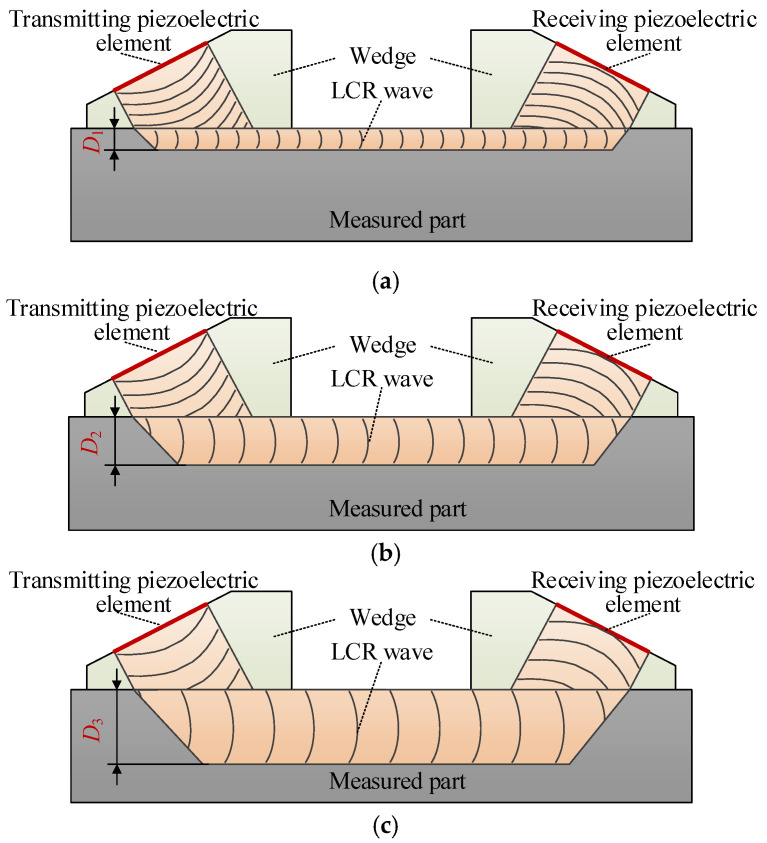
Ultrasonic transducers with different frequencies can generate LCR waves propagating at different depths in the material. (**a**) Propagation depth of LCR wave at *f*_1_ frequency; (**b**) propagation depth of LCR wave at *f*_2_ frequency; (**c**) propagation depth of LCR wave at *f*_3_ frequency.

The propagation depth of the LCR wave in the measured medium is related to the excitation frequency of the ultrasonic transducer, as shown in Figure 3. In the figure, the excitation frequency relationship of the ultrasonic transducer is *f*_1_ > *f*_2_ > *f*_3_. According to the existing research, the relationship between the propagation depth of the LCR wave is *D*_1_ < *D*_2_ < *D*_3_ [25].

The LCR waves propagating at different depths below the material surface can be obtained by changing the excitation frequency of the transducer. Then, according to the influence of stress at different depths of LCR wave velocity and the correlation model between stress and wave velocity, the stress distribution at different depths below the material surface can be finally determined.

## 3. The Finite Element Calculation Model

### 3.1. Numerical Simulation Method for LCR Waves

When there is stress in the material, pre-strain $\varepsilon_{IJ}^{i}$ will occur. Based on the stress–strain relationship in isotropic solids, the pre-strain calculation expression is:

(2) $$\{\begin{array}{l} \varepsilon_{IJ}^{i}=\frac{1+v}{E}\sigma_{IJ}-\delta_{IJ}\frac{v}{E}\sigma_{KK}\\ E=\frac{\mu (3\lambda +2\mu )}{\left(\lambda +\mu \right)}\\ v=\frac{\lambda }{2(\lambda +\mu )} \end{array}$$

where *E* and *v* are the elastic modulus and Poisson’s ratio of the material, respectively. *λ* and *μ* are lame constants.

According to Equation (2), the stiffness coefficient matrix under stress can be obtained. According to the finite element numerical calculation method, Equation (1) can be calculated by solving the discrete wave motion, Equation (3):

(3) $$\left[M]\{\ddot{U}\}+[C]\{\dot{U}\}+[K]\{U\}=\{F\right\}$$

where [*M*] is the mass matrix and $[M]=\sum_{e}\left[M^{e}\right]$. [*C*] is the damping matrix and $[C]=\sum_{e}\left[C^{e}\right]$. [*K*] is the stiffness matrix and $[K]=\sum_{e}\left[K^{e}\right]$. {*U*} is the node displacement and {*F*} is the stress vector. The matrices are processed as follows:

(4) $$\left\{ \begin{array}{l} \;\left[M^{e}\right]=\rho \int_{V^{e}}{\left[N\right]}^{T}[N]dV\\ \;\left[C^{e}\right]=\gamma \int_{V^{e}}{\left[N\right]}^{T}[N]dV\\ \;\left[K^{e}\right]=\int_{V^{e}}{\left[B\right]}^{T}\left[C_{IJKL}\right][B]dV \end{array}\right.$$

where [*N*] and [*B*] are shape functions and the strain matrix, respectively.

Equation (4) can be solved by the following explicit difference Equation [29]:

(5) $$\{\begin{array}{l} {\left\{\dot{U}\right\}}_{t+\Delta t}={\left\{\dot{U}\right\}}_{t-\Delta t}-2\Delta t{\left[M\right]}^{-1}[C]{\left\{\dot{U}\right\}}_{t}-2\Delta t{\left[M\right]}^{-1}[K]{\left\{U\right\}}_{t}+2\Delta t{\left[M\right]}^{-1}\{F\}\\ {\left\{U\right\}}_{t+\Delta t}={\left\{U\right\}}_{t}+\frac{{\left\{\dot{U}\right\}}_{t+\Delta t}+{\left\{\dot{U}\right\}}_{t}}{2}\Delta t \end{array}$$

### 3.2. Geometric Model

**Figure 4:**
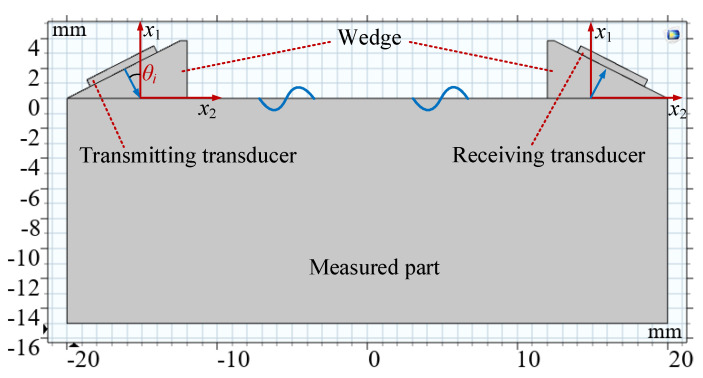
Geometric model in simulation calculation.

Two transducers were used for one send and one receive. The materials of the wedge and the measured part are PMMA and 1045 steel, respectively. According to Snell’s law, the first critical angle *θ_i_* = 27.23°, as shown in Figure 4. Therefore, the transmitting and receiving angles are both 27.23° in the finite element simulation.

The length and thickness of the measured part in the model are 40 and 15 mm, respectively. The ultrasonic transducer was installed in the center of the inclined plane of the PMMA wedge. The diameter of the piezoelectric elements of the transmitting and receiving transducers are 5 mm.

### 3.3. Calculation Parameters

The ultrasonic excitation signal is described by the following formula:

(6) $$S\left(t\right)=100\exp\left(-{\left(\left(t-2T_{0}\right)/\left(T_{0}/2\right)\right)}^{2}\right)\sin\left(2\pi ft\right)$$

where *f* is the ultrasonic excitation frequency, *T*_0_ is the period of the excitation signal and *t* is the time.

**Figure 5:**
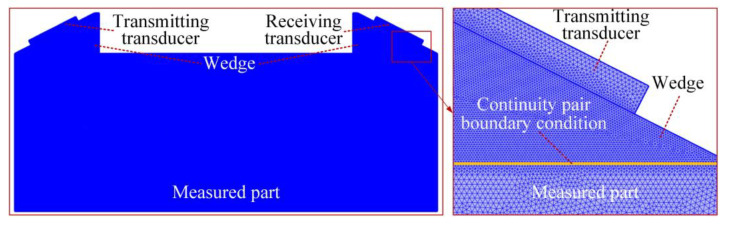
Finite element mesh in simulation calculation.

In this paper, the commercial simulation software COMSOL (version 5.5) was used for finite element calculation. In order to control the accuracy of the finite element calculation, the mesh size was set to 1/20 of the wavelength, and the mesh type is the triangle, as shown in Figure 5. In the time-varying analysis, the time step was set to 0.1 ns (i.e., 1 × 10^−10^ s) to accurately extract the change of the ultrasonic signal in the propagation time.

## 4. Analysis of Finite Element Calculation Results

### 4.1. Generation and Propagation Process of LCR Wave

**Figure 6:**
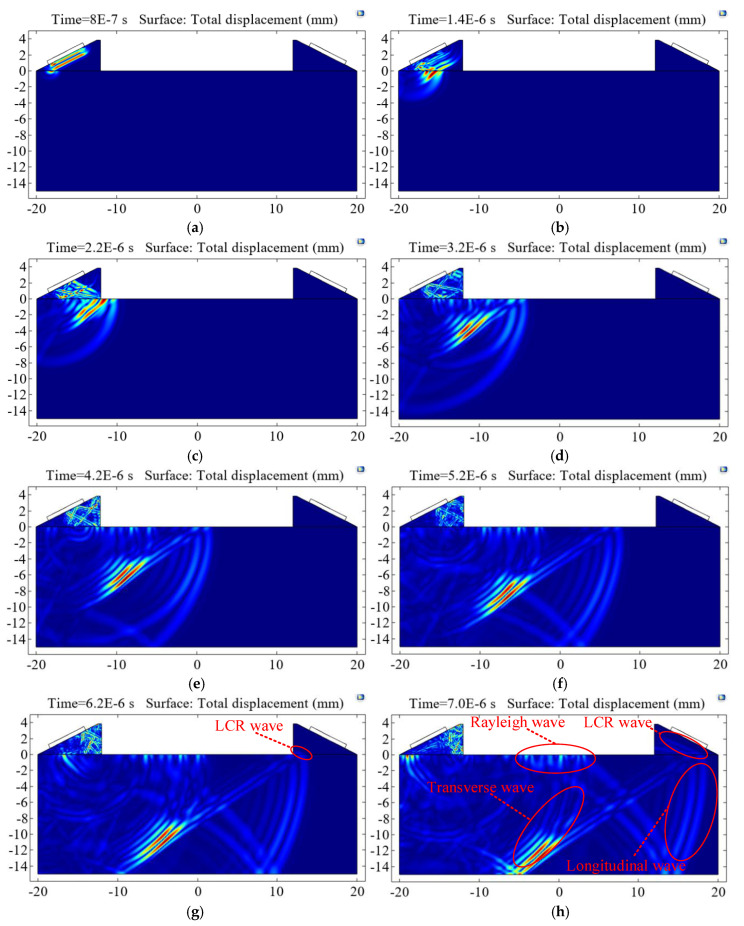
The generation and propagation process of LCR wave in time (**a**) *t* = 8E-7 s, (**b**) *t* = 1.4E-6 s, (**c**) *t* = 2.2E-6 s, (**d**) *t* = 3.2E-6 s, (**e**) *t* = 4.2E-6 s, (**f**) *t* = 5.2E-6 s, (**g**) *t* = 6.2E-6 s, and (**h**) *t* = 7.0E-6 s.

The longitudinal wave was excited on the surface of the wedge and propagates into the steel with the first critical angle of 27.23°. Then longitudinal waves, transverse waves, surface waves (Rayleigh waves), and LCR waves were generated in the steel. LCR waves arrived at the receiving transducer first, and the process is shown in Figure 6.

It can be seen from Figure 6 that the ultrasonic wave already appeared under the wedge at 0.8 μs. At 1.4 μs, an arc-shaped wave appeared, which was the longitudinal wave propagating in the steel. At 3.2 μs, an approximately diagonal wave appeared behind the longitudinal wave, which is called the head wave. The other circular wave that appeared behind the head wave is the slower transverse wave. One end of the head wave was tangent to the transverse wavefront, and the other end was connected to the longitudinal wave on the surface of the measured part. At 6.2 μs, the LCR wave began to reach the receiving wedge. At 7 μs, the LCR wave propagated from the measured part to the wedge and was clearly seen to be parallel to the slope. This wave gradually propagated in the wedge and finally reached the receiving transducer. 

The longitudinal wave propagated along the surface of the measured part and continuously underwent mode conversion at the interface to generate the transverse wave. The transverse wave envelopes generated at different distances formed the so-called head wave, which is one of the factors of LCR wave formation.

**Figure 7:**
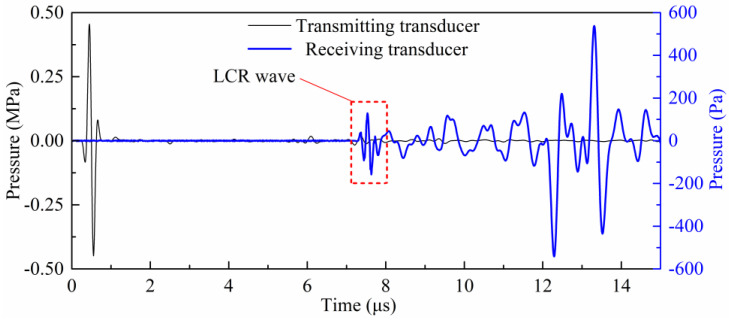
The sound pressure signals of the transmitting and receiving transducers.

The time characteristics of the sound pressure signals of the transmitting and receiving transducers are shown in Figure 7. It can be seen in the figure that the LCR wave has the fastest propagation velocity and reached the receiving transducer first, which is consistent with the actual situation.

In Figure 7, the receiving time of the first trough of the LCR wave is 7449.1 ns. To verify this simulation calculation result and ensure the accuracy of the subsequent simulation analysis of the LCR wave propagation law, the simulation calculation result was compared with the experimental data. In the experiment, the same wedge material and measured material as in the simulation were used, and the propagation path of the LCR wave is also consistent with the simulation. The experiment shows that the receiving time of the first trough of the LCR wave was 7557.3 ns. Converting the receiving time to the velocity of sound, the relative error of the simulation calculation is 1.85%. Therefore, the numerical scheme adopted in this paper is correct and feasible.

### 4.2. Determination Method of LCR Wave Propagation Depth

**Figure 8:**
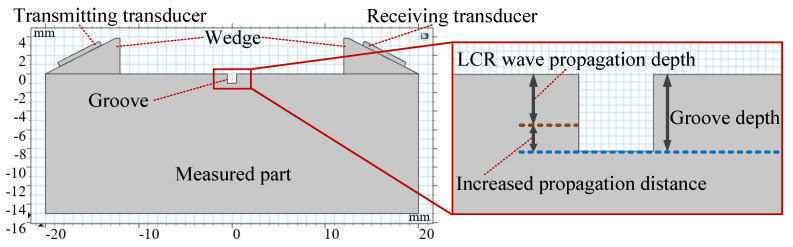
The method of determining the propagation depth of LCR waves.

**Figure 9:**
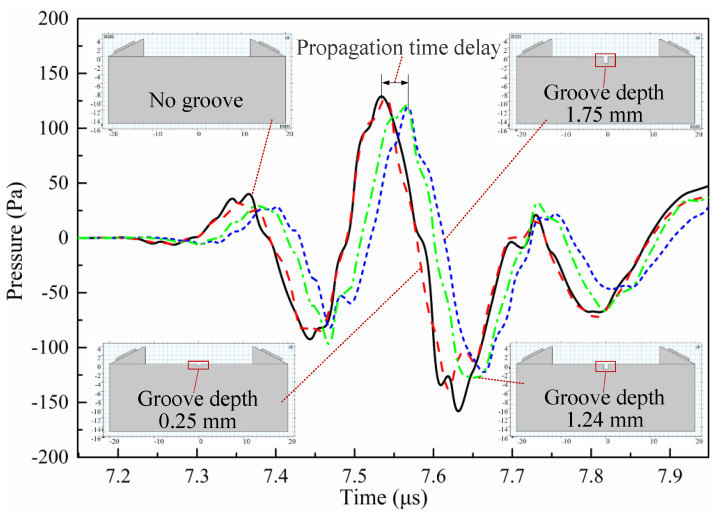
Time extension of LCR wave signal with a frequency of 4 MHz at different groove depths.

As shown in Figure 8, a groove with a width of 1 mm was set on the surface of the measured part. The LCR wave will be hindered when it propagates to the groove, resulting in a time extension to the receiving transducer. When the depth of the groove is far less than the propagation depth of LCR wave, the blocking effect of the groove is very small, and the propagation time extension can be ignored. In this case, the influence of the groove on the LCR wave is mainly reflected in the slight decrease in amplitude. If the groove depth is greater than the propagation depth of the LCR wave, the propagation distance of the LCR wave will be increased—that is, the LCR wave will propagate downward for a certain distance, which will cause the LCR wave propagation time extension to increase significantly, as shown in Figure 9. Then, the depth of the groove needs to be changed to simulate several times. When the propagation time extension is equal to the time *Δt* required for LCR wave to propagate 1/20 of its own wavelength, the depth is considered as the propagation depth of the LCR wave.

Take the method of determining the propagation depth of LCR waves with a frequency of 4 MHz as an example. Firstly, the propagation process of the LCR wave without a groove was simulated to obtain its signal data. Secondly, the propagation process of the LCR wave when the groove depth was *d*_1_ was simulated and its signal data were obtained. Thirdly, the cross-correlation operation was performed on the signal data of the LCR wave with or without grooves to calculate the propagation time extension *Δt*_1_ caused by the groove with the depth of *d*_1_. Finally, the comparison between *Δt*_1_ and the judgment basis *Δt* was made. If *Δt*_1_ is less than the judgment basis, the groove depth needs to be increased to simulate again. If *Δt*_1_ is greater than the judgment basis, the groove depth will be reduced. The simulation was carried out by changing the groove depth several times until *Δt_i_* and *Δt* were equal. The propagation depth of an LCR wave with a frequency of 4 MHz was obtained as *d_i_* = 1.24 mm.

### 4.3. Correlation Model of LCR Wave Propagation Depth and Frequency

**Figure 10:**
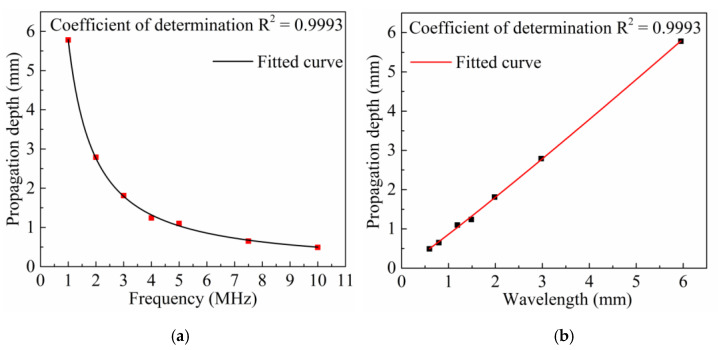
Relationship between the LCR wave propagation depth and frequency (**a**) and wavelength (**b**).

Referring to the step of determining the propagation depth of an LCR wave with a frequency of 4 MHz, the propagation simulation of LCR waves with frequencies of 1, 2, 3, 5, 7.5, and 10 MHz was carried out successively. The propagation depths were 5.78, 2.79, 1.81, 1.10, 0.65, and 0.49 mm, respectively, as shown in Figure 10a. According to the distribution characteristics of the propagation depth of LCR waves at different frequencies, exponential function and power function were used to fit the LCR wave frequency and its corresponding propagation depth, respectively. The coefficients of determination for exponential function and power function fitting are 0.9901 and 0.9993, respectively, so the power function fitting was used to obtain the calculation formula (7) of the propagation depth of LCR waves at different frequencies:

(7) $$D=C_{1}f^{-1.07}$$

where *f* is the frequency of the LCR wave, MHz. C_1_ = 5.79 is the dimensionless coefficient. *D* is the propagation depth of LCR wave (mm).

From formula (7), it can be concluded that the propagation depth *D* of the LCR wave is inversely proportional to the frequency *f*; that is, the propagation depth of the wave decreases as the frequency increases. Therefore, according to formula (7), the propagation depth of the LCR wave can be accurately controlled by changing the frequency, and then the accurate stress distribution at different depths can be obtained.

Formula (7) reflects the relationship between LCR wave propagation depth and frequency. According to the calculation formula between wavelength and frequency, the propagation depth of LCR waves at different wavelengths can be obtained, as shown in Figure 10b. According to the distribution characteristics of LCR wave propagation depths at different wavelengths, the wavelength and corresponding propagation depth of LCR waves are fitted by using the linear relationship and power function, respectively. The coefficients of determination of the linear relationship and the power function fitting are 0.9991 and 0.9993, respectively, so the power function fitting is used to obtain the calculation (formula (8)) of the propagation depth of LCR waves at different wavelengths:

(8) $$D=C_{2}\lambda^{1.07}$$

where C_2_ = 0.86 is the dimensionless coefficient, λ is the wavelength of LCR wave (mm).

In order to verify the correctness of the simulation results, the experimental parameters in reference [30] are substituted into the LCR wave propagation depth model obtained in this paper, and the experimental results and theoretical calculation values are compared and analyzed. In reference [30], the authors studied the propagation depth of LCR waves by milling grooves with different depths on the measured part made of 304L stainless steel. The results show that the propagation depth of the LCR wave is 1 mm when the frequency is 5 MHz. The propagation velocity of the longitudinal wave in 304L is 5640 m/s, so the wavelength of the LCR wave is 1.128 mm when the frequency is 5 MHz. Substituting it into Equation (8), the propagation depth was calculated to be 0.98 mm. Therefore, the difference between the two methods is 0.02 mm.

The reasons for the difference of 0.02 mm between the calculation model and the experimental results in reference [30] mainly include the following aspects: In terms of experiments, first of all, there stress may be concentrated around the machined groove, and the propagation velocity of ultrasonic waves will be affected by the stress. Tensile stress will slow down its propagation velocity, while compressive stress will accelerate the wave propagation velocity. Therefore, the stress will lead to a change of wave propagation time and affect the judgment of the LCR wave propagation depth. Secondly, in the process of measuring the propagation depth of LCR waves by changing the groove depth, it is necessary to install ultrasonic transducers repeatedly. Therefore, it is inevitable to change the coupling state and the thickness of the coupling layer, resulting in the propagation time difference of the LCR wave. Thirdly, due to the existence of machining error, there is a deviation between the actual groove depth and the design value, which affects the measurement accuracy of LCR wave propagation depth. In terms of the calculation model, the dimensionless coefficient in formula (8) is suitable for the 1045 steel material simulated in this paper. If this formula is generalized and applied, this coefficient needs to be corrected; that is, corresponding correction coefficients should be given for different materials.

## 5. Discussions

In addition to the influence of frequency, whether the propagation depth of LCR wave is related to other factors, such as the material of wedge, the diameter of the piezoelectric element and the value of excitation voltage, etc., remains to be studied. Therefore, in this section, the influence of the wedge material, piezoelectric element diameter, and excitation voltage on the propagation depth of LCR waves is studied by changing relevant parameters. In addition, the significant challenges in the measurement of the stresses of rotors with complex geometries are also analyzed in this section.

### 5.1. Effect of Wedge Material on Propagation Depth of LCR Waves

The LCR wave was generated by the longitudinal ultrasonic wave incident on the measured part from the wedge at the first critical angle. Therefore, it is necessary to calculate the first critical angle by Snell’s law and design the wedge to make the ultrasonic longitudinal wave incident on the measured part at the first critical angle. The wedge material should have the properties of a low sound velocity, small sound attenuation coefficient, and easy processing. PMMA and PS are two commonly used wedge materials.

**Figure 11:**
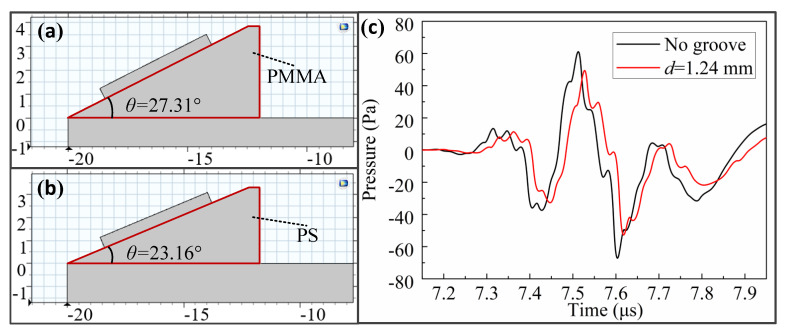
Analysis of the effect of wedge material on the LCR wave propagation depth: (**a**) the wedge material is Polymethyl methacrylate (PMMA); (**b**) the wedge material is polystyrene (PS); (**c**) propagation time extension caused by 1.24 mm groove when wedge material is PS.

In this paper, PMMA was used as the wedge material in the simulation analysis of the relationship between the propagation depth of LCR wave and frequency, as shown in Figure 11a. To study the influence of wedge material on the propagation depth of LCR waves, PS was selected for the simulation and comparative analysis. According to Snell’s law, the first critical angle is 23.16° when using PS as the wedge, as shown in Figure 11b. When using PMMA as the wedge material, the LCR propagation depth at a frequency of 4 MHz is 1.24 mm. Therefore, as shown in Figure 11c, we directly analyzed the amount of LCR wave propagation time extension caused by the 1.24 mm groove when the PS was used as a wedge. By comparing this extension with that when using PMMA as the wedge, it can be determined as to whether the wedge material affects the propagation depth of LCR waves.

Using the cross-correlation algorithm, it can be seen that when the PS was used as the wedge, the propagation time extension of the LCR wave generated by 1.24 mm groove is 12.60 ns. When using PMMA, it is 12.51 ns. Therefore, the change rate of the propagation time extension after changing the wedge material is only 0.72%. It can be considered that the propagation depth of the LCR wave with a frequency of 4 MHz when using PS as the wedge is still 1.24 mm; that is, the propagation depth of the LCR wave does not change after the wedge material is changed.

By comparing Figure 9 and Figure 11c, it can be concluded that the amplitude of the LCR wave when using PS as the wedge is smaller than that using the PMMA. This is because the acoustic impedance of PMMA is greater than that of PS, so the reflectivity of the PMMA–steel interface is less than that of the PS–steel interface. The low reflectivity of ultrasonic waves means that the energy loss is small.

### 5.2. Effect of Piezoelectric Element Diameter on Propagation Depth of LCR Wave

According to the stress measurement model based on the LCR wave in Section 2, the diameter of the piezoelectric element of the ultrasonic transducer determines the spatial resolution of the stress measurement. The smaller the diameter of the piezoelectric element, the smaller the measurement area, so the higher the spatial resolution of the stress measurement. Conversely, if the diameter of the piezoelectric element is larger, it means that the spatial resolution of the stress measurement is lower.

**Figure 12:**
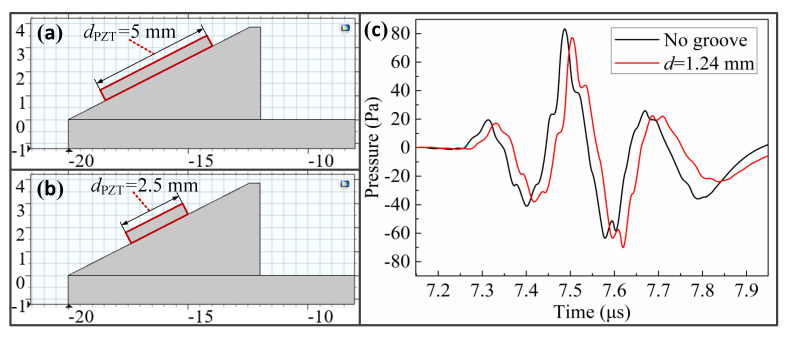
Analysis of the effect of piezoelectric element diameter on propagation depth of LCR wave: (**a**) piezoelectric element diameter is 5 mm; (**b**) piezoelectric element diameter is 2.5 mm; (**c**) propagation time extension caused by 1.24 mm groove when piezoelectric element diameter is 2.5 mm.

In this paper, the diameter of the piezoelectric element used in the simulation study of the relationship between the propagation depth of LCR wave and the frequency was 5 mm, as shown in Figure 12a. To study the effect of piezoelectric element size on the propagation depth of LCR, a piezoelectric element with a diameter of 2.5 mm was selected for simulation and comparative analysis, as shown in Figure 12b. When using a piezoelectric element with a diameter of 2.5 mm, the LCR wave propagation time extension caused by the 1.24 mm groove was directly analyzed, as shown in Figure 12c. By comparing this extension with the extension when the diameter of the piezoelectric element was 5 mm, it can be determined whether the piezoelectric element diameter affects the propagation depth of LCR wave.

Using the cross-correlation algorithm, it can be seen that when the 2.5 mm diameter piezoelectric element was used, the propagation time extension of the LCR wave generated by the 1.24 mm groove is 12.56 ns. The difference is 0.05 ns compared to when using a piezoelectric element with a diameter of 5 mm. Therefore, the change rate of the propagation time extension after changing the diameter of the piezoelectric element is only 0.40%. It can be considered that the propagation depth of LCR waves with a frequency of 4 MHz is still 1.24 mm when the piezoelectric element with a diameter of 2.5 mm is used; that is, the propagation depth of LCR waves does not change after the change of piezoelectric element diameter.

By comparing Figure 9 and Figure 12c, it can be concluded that the sound pressure amplitude of the LCR wave when using a piezoelectric element with a diameter of 2.5 mm is smaller than that using a piezoelectric element with a diameter of 5 mm. This is because the sound pressure amplitude is positively related to the diameter of the piezoelectric element. The larger the diameter of the piezoelectric element, the larger the sound pressure amplitude of the LCR wave.

### 5.3. Effect of Excitation Voltage on the Propagation Depth of LCR Waves

There is a positive correlation between the sound pressure amplitude of the LCR wave and the excitation voltage of the ultrasonic transducer, so the corresponding excitation voltage can be used according to the measurement requirements. In order to study whether different excitation voltages will affect the propagation depth of the LCR wave and thus affect the spatial range of stress measurements, it is necessary to carry out the corresponding simulation and comparative analysis.

**Figure 13:**
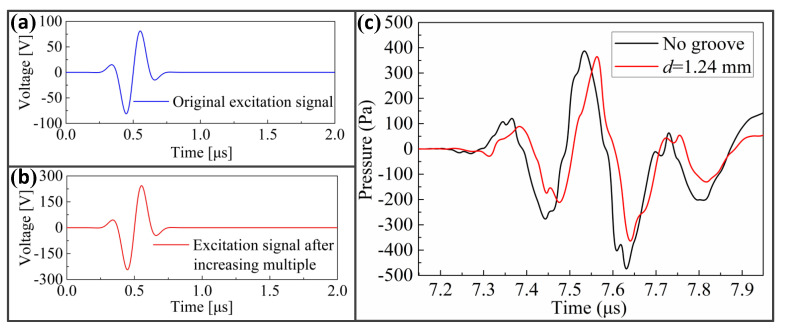
Analysis of the effect of excitation voltage on propagation depth of LCR wave: (**a**) excitation signal *S*_1_(*t*); (**b**) excitation signal *S*_2_(*t*); (**c**) propagation time extension caused by 1.24 mm groove when the excitation signal is *S*_2_(*t*).

In this paper, the excitation signal $S_{1}\left(t\right)=100\exp\left(-{\left(\left(t-2T_{0}\right)/\left(T_{0}/2\right)\right)}^{2}\right)\sin\left(2\pi ft\right)$ was used in the simulation study of the relationship between the propagation depth of LCR wave and frequency, as shown in Figure 13a. To study the effect of the excitation voltage value on the propagation depth of the LCR wave, $S_{2}\left(t\right)=300\exp\left(-{\left(\left(t-2T_{0}\right)/\left(T_{0}/2\right)\right)}^{2}\right)\sin\left(2\pi ft\right)$ was selected for simulation and comparative analysis, as shown in Figure 13b. *S*_2_(*t*) has the same waveform as *S*_1_(*t*), and its value is three times that of *S*_1_(*t*). When *S*_2_(*t*) was used as the excitation signal, the propagation time extension of the LCR wave caused by a 1.24 mm groove was analyzed directly, as shown in Figure 13c. By comparing this extension with that when the excitation signal *S*_1_(*t*) was used, it can be judged whether the excitation voltage influences the propagation depth of the LCR wave.

Using the cross-correlation algorithm, it can be seen that when the excitation signal *S*_2_(*t*) was used, the propagation time extension of LCR wave generated by the 1.24 mm groove is 12.51 ns. It is the same as that when the excitation signal *S*_1_(*t*) was used. Therefore, when the excitation signal *S*_2_(*t*) was used, the propagation depth of the LCR wave with a frequency of 4 MHz is still 1.24 mm; that is, the propagation depth does not change after the excitation voltage is increased. By comparing Figure 9 and Figure 13c, it can be concluded that the sound pressure amplitude of the LCR wave generated by the excitation signal *S*_2_(*t*) is exactly three times that generated by *S*_1_(*t*). This means that as the value of the excitation signal increases, the sound pressure amplitude also increases proportionally.

### 5.4. Significant Challenges in Stress Measurement of Rotors with and Complex Geometries

Modern aero-engine rotors generally have complex geometries such as a curved surface, which brings great challenges to rotor stress measurements based on LCR waves. First of all, the curvature of the rotor surface will cause the ultrasonic beam to scatter and focus, so how to accurately and reliably project the ultrasonic beam to the area to be measured is the primary challenge in the stress measurement of complex structures. Secondly, changes in curvature lead to changes in the profile of the measured surface. Therefore, the mechanical mechanism needs to adjust and control the attitude of the transducers in real time, so that the transducers can track the contour during the measurement process to ensure an accurate transmission and reception of acoustic signals. Finally, the commonly used piezoelectric elements have a certain size (diameter 6–12 mm), so when the rotor surface curvature is large, it is more difficult to arrange the transducer.

## 6. Conclusions

According to the wave equation and Snell’s law, the propagation model of the LCR wave in the measured material is established. The whole process from excitation to receiving of LCR wave by a piezoelectric element with a diameter of 5 mm in 40 × 15 mm steel is simulated. By analyzing this process, the mechanism of LCR wave generation is intuitively explained, which will also help to further the study of LCR waves. The amount of propagation time extension is used to quantitatively characterize the blocking effect of grooves of different depths on the wave, and then the propagation depth of LCR waves of different frequencies is determined. A theoretical formula to characterize the propagation depth of LCR waves is proposed. Finally, the effect of the wedge material, piezoelectric element diameter, and excitation voltage on the propagation depth of LCR waves are comprehensively considered, which provides a reference for the selection of related devices and parameters in actual measurement. Through the LCR wave propagation depth determination method and theoretical formula proposed in this paper, the law of LCR wave propagation depth in specific materials can be determined more accurately and quickly. In this way, the propagation depth of the LCR wave can be precisely controlled by changing the excitation frequency. The results obtained according to the method proposed in this paper have been used to guide the measurement of stress at different depths of aero-engine rotor components based on the acoustoelastic effect. This study can provide valuable theoretical and technical reference for stress measurement at different depths in the production process and subsequent assembly state of aero-engines and other large high-speed rotating equipment such as ship and power station gas turbines.
